# Probiotics supplement in children with severe hand, foot, and mouth disease: A systematic review and meta-analysis protocol

**DOI:** 10.1097/MD.0000000000017939

**Published:** 2019-11-11

**Authors:** Haibo Zhang, Lei Zhang, Jiadong Xie, Wenting Wen, Luxia Wei, Bo Nie

**Affiliations:** a Nanjing University of Chinese Medicine, Nanjing, China; b University College London, London, England.

**Keywords:** hand foot and mouth disease, meta-analysis, probiotics, systematic review

## Abstract

**Background:**

Severe hand, foot, and mouth disease (HFMD) is an acute infectious disease caused by infection with serotypes of Enterovirus A, most commonly by enterovirus A71 and coxsackievirus A16. Clinical symptoms usually include fever, malaise, rashes on hands and feet, and oral vesicles. Of note, severe and even life-threatening complications can develop rapidly in young children, such as acute pulmonary edema, cardiopulmonary failure, aseptic meningitis, encephalitis and acute flaccid paralysis. Probiotics supplement have been demonstrated play a positive role as a therapeutic approaches for modulation of gut microbiota. This study aims to systematically investigate the efficacy and safety of probiotics for children with severe HFMD.

**Methods:**

All randomized controlled trials related to probiotics and severe HFMD will be searched in 9 electronic databases (PubMed, Cochrane Library, Embase, ClinicalTrails, China National Knowledge Infrastructure, Sino Med, ScienceDirect, VIP, and Wanfang Data databases) from their inception to November 2019. The primary outcome is total effective rate, fever clearance time, rash regression time, remission time of neurological symptoms, and clinical cure time. Two researchers will perform the study selection, data extraction, and assessment of risk of bias independently. RevMan software (version 5.3) will be used for data synthesis.

**Results:**

The findings of this study will be published in a peer-reviewed journal.

**Conclusion:**

The study will provide evidence to judge whether probiotics is an effective therapeutic intervention for severe HFMD.

**PROSPERO registration number:**

PROSPERO CRD42019152946.

## 1 Introduction

Hand, foot, and mouth disease (HFMD) is an infectious gastrointestinal disease with characteristic features of fever, oral ulcers, and vesicular rashes on the hands, feet, and buttocks,^[1]^ caused primarily by human enterovirus-A71 (EV-A71) and coxsackievirus-A16 (CV-A16),^[2]^ and mostly affects children.^[3]^ The majority of HFMD cases are mild or typically asymptomatic, but severe and potentially life-threatening central nervous system (CNS) complication such as encephalitis, meningitis, acute flaccid paralysis (AFP), myocarditis, and pulmonary edema have also been reported.^[4–6]^ According to the national infectious disease direct network reports from China, from the year 2010 to 2012, the average annual incidence of HFMD was estimated 0.12%, about 1.1% of which developed into severe cases, and the mortality of HFMD was 0.03%.^[7]^ Although the inactivated EV71 vaccine has been widely used to vaccinate infants and young children in several provinces of mainland China and has shown to be effective at protecting them from EV71-associated severe HFMD, the number of severe cases is still large, posing a threat to infants and young children in rural areas of Asia-Pacific region.^[3,8–10]^

In recent years, the effect of probiotics on severe HFMD has raised concern, and some clinical randomized controlled trials (RCTs) have been performed to observed the treatment effect. Most trials have concluded that probiotics is efficient and, at least in part, beneficial biochemical criteria. Considering the uncompleted and contradictory results from clinical RCTs, the effect of probiotics on severe HFMD needs further discuss. The purpose of this systematic review and meta-analysis is to systematically evaluate the efficacy and safety of probiotics on children with severe HFMD.

## 2 Methods

### 2.1 Inclusion criteria for study selection

#### 2.1.1 Types of studies

All randomized controlled trials (RCTs) evaluating the use of probiotics in the treatment of children with severe HFMD will be included without language limitations.

#### 2.1.2 Types of participants

Standard diagnosis of severe HFMD children will be included in analysis, regardless of their gender, ethnicity, and background, according to the clinical guidelines.

#### 2.1.3 Types of interventions

In this study, the intervention received by children with severe HFMD in the experimental group will be probiotics, while the control group will be administered other therapy or placebo.

#### 2.1.4 Outcomes

Primary outcomes are the differences between probiotic and control groups immediately after treatment in:

1. Total effective rate;
2. Fever clearance time;
3. Rash regression time;
4. Clinical cure time;
5. Remission time of neurological symptoms;
6. Incidence of adverse effects.

### 2.2 Search strategy

#### 2.2.1 Electronic searches

Nine databases (PubMed, Cochrane Library, Embase, ClinicalTrails, China National Knowledge Infrastructure, Sino Med, ScienceDirect, VIP, and Wanfang Data databases) will be searched from their inception to November 2019 without language restrictions. Two reviewers (HZ and LZ) will independently search the studies. Any differences will be resolved through discussion with a third author (WW).

Search strategy of PubMed was as follows:

1. probiotics
2. (hand, foot and mouth disease) OR HFMD
3. Step 1 AND step 2.

#### 2.2.2 Searching other resources

Potential eligible studies will be searched for relevant conference proceedings and reference lists of previously published reviews.

### 2.3 Data collection and analysis

#### 2.3.1 Study selection

The selection process will be summarized according to PRISMA flow diagram. Two reviewers (HZ and LZ) will independently search and evaluate every relevant study according to the Cochrane Handbook. They will screen the retrieved literaturebyreading the titles and abstracts. The full text of relevant literature will then be read, and the studies will be selected in accordance with the inclusion criteria. Any disagreements will be resolved by discussion with the third reviewer (WW).

#### 2.3.2 Data extraction

Two reviewers (HZ and JX) will use a data extraction form to extract data on participants, randomization, interventions, outcomes, duration, follow-up, reasons for discontinuation, number of treatment-related adverse events, author information, and conflicts of interest. Another reviewer (LZ) will double check the extracted data. Any discrepancies should be resolved by negotiation between the 2 reviewers with the help of a third author (WW).

#### 2.3.3 Processing missing data

When experimental data are missing or inadequate, we will attempt to contact the original author of the study by e-mail or telephone to obtain sufficient and comprehensive data. Incomplete data will be discarded if sufficient data cannot be retrieved.

#### 2.3.4 Risk of bias assessment

Two reviewers (HZ and JX) will evaluate the methodological quality of the including studies using the Cochrane Collaboration's tool. Each study will be evaluated for validity based on the following 7 aspects: random sequence generation, allocation concealment, blinding of participants and personnel, blinding of outcome assessment, incomplete data assessment, selective outcome reporting, and other sources of bias.

#### 2.3.5 Heterogeneity assessment and statistical analysis

Review Manager 5.3 software will be utilized for statistical analysis on the basis of homogeneity of the included trials. Weighted mean differences (WMDs) and 95% CIs will be used for analysis when units are the same, while standardized mean differences (SMDs) and 95% CIs will be used when units are different. The *I*^2^ statistic will be utilized for assessing the heterogeneity of the included trials. A fixed-effect model will be applied to calculate the pooled statistics in the absence of substantial heterogeneity (*I*^2^ < 50% and *P* ≥ .1). Conversely, if statistical heterogeneity is identified (*I*^2^ > 50% or *P* < .1), the causes of the heterogeneity will be identified first by subgroup analysis. If the heterogeneity cannot be readily explained, a random effects model will be interpreted with caution.

#### 2.3.6 Subgroup analysis

If significant heterogeneity is observed in the included studies, subgroup analysis will be performed based on interventions, controls, and outcome measurements.

#### 2.3.7 Assessment of reporting biases

If there are a sufficient number of articles (>10) included under the same endpoint addressing the same question, a funnel plot will be used to measure publication bias.

#### 2.3.8 Sensitivity analysis

If sufficient test data are available, sensitivity analysis will be performed to determine whether the conclusion is robust.

#### 2.3.9 Grading of quality of evidence

The Grading of Recommendations Assessment, Development, and Evaluation will be utilized for assessing the quality of evidence for the main outcomes. The quality of evidence will be categorized as high, moderate, low, or very low.

#### 2.3.10 Ethics and dissemination

Ethics approval is not required because the data will not include individual patient data and, therefore, will not incur raise any privacy issues. The results of this systematic review will be disseminated only in a peer-reviewed publication.

## 3 Discussion

Severe HFMD is an implication of many risk factors.^[1]^ Previous studies have confirmed the role of dysbiosis of the gut microbiota in severe HFMD, and probiotics could be beneficial for children with severe HFMD. This study aims to systematically investigate the efficacy and safety of the probiotics for children with severe HFMD. The findings will provide further evidence for the management of severe HFMD.

## Author contributions

**Conceptualization:** Wenting Wen, Luxia Wei, Bo Nie.

**Data curation:** Wenting Wen, Haibo Zhang, Lei Zhang, Jiadong Xie.

**Formal analysis:** Wenting Wen, Haibo Zhang, Lei Zhang, Jiadong Xie.

**Funding acquisition:** Jiadong Xie, Luxia Wei.

**Writing – original draft:** Wenting Wen, Haibo Zhang.

**Writing – review & editing:** Luxia Wei.

Wenting Wen orcid: 0000-0002-3930-818X.
